# Beyond HRD Status: Unraveling Genetic Variants Impacting PARP Inhibitor Sensitivity in Advanced Ovarian Cancer

**DOI:** 10.1158/2767-9764.CRC-24-0294

**Published:** 2024-12-26

**Authors:** Maj K. Kjeldsen, Morten Jørgensen, Dina Sofie B. Grønseth, Martin Schønemann-Lund, Gitte-Bettina Nyvang, Charlotte Aaquist Haslund, Anja Oer Knudsen, Anne Krejbjerg Motavaf, Susanne Malander, Maarit Anttila, Gabriel Lindahl, Johanna Mäenpää, Maria Dimoula, Theresa L. Werner, Trine Zeeberg Iversen, Sakari Hietanen, Lars Fokdal, Hanna Dahlstrand, Line Bjørge, Michael J. Birrer, Mansoor R. Mirza, Maria Rossing

**Affiliations:** 1 Department of Oncology, Copenhagen University Hospital, Rigshospitalet, Copenhagen, Denmark.; 2 Center for Genomic Medicine, Copenhagen University Hospital, Rigshospitalet, Copenhagen, Denmark.; 3 Nordic Society of Gynecological Oncology-Clinical Trial Unit (NSGO-CTU), Copenhagen, Denmark.; 4 Department of Anesthesia and Intensive Care, Odense University Hospital, Odense, Denmark.; 5 Department of Oncology, Odense University Hospital, Odense, Denmark.; 6 Department of Oncology, Aalborg University Hospital, Aalborg, Denmark.; 7 Department of Oncology, Skane University Hospital, Lund University, Lund, Sweden.; 8 Department of Clinical Science, Skane University Hospital, Lund University, Lund, Sweden.; 9 Department of Obstetrics and Gynecology, Kuopio University Hospital, Kuopio, Finland.; 10 Department of Oncology, Linköping University, Linköping, Sweden.; 11 Department of Biomedical and Clinical Sciences, Linköping University, Linköping, Sweden.; 12 Tampere University and Cancer Center, Tampere University Hospital, Tampere, Finland.; 13 Department of Oncology, Uppsala University Hospital, Uppsala, Sweden.; 14 Huntsman Cancer Institute, University of Utah, Salt Lake City, Utah.; 15 Department of Oncology, Herlev-Gentofte University Hospital, Herlev, Denmark.; 16 Department of Obstetrics and Gynecology, Turku University Hospital and FICAN West, Turku, Finland.; 17 Department of Oncology, Vejle Hospital, University Hospital of Southern Denmark, Odense, Denmark.; 18 Department of Oncology-Pathology, Karolinska Institutet and Theme Cancer, Karolinska University Hospital, Stockholm, Sweden.; 19 Department of Obstetrics and Gynecology, Haukeland University Hospital, Bergen, Norway.; 20 Department of Clinical Science, Centre for Cancer Biomarkers CCBIO, University of Bergen, Bergen, Norway.; 21 Winthrop P. Rockefeller Cancer Institute, Little Rock, Arkansas.; 22 Department of Clinical Medicine, Faculty of Health and Medical Sciences, University of Copenhagen, Copenhagen, Denmark.

## Abstract

The management of advanced epithelial ovarian cancer (AOC) has undergone significant advancements with the emergence of molecular diagnostics, particularly in predicting responses to PARP inhibitors (PARPi) based on homologous recombination deficiency (HRD) status. However, understanding sensitivity and resistance beyond HRD status remains elusive. This study aims to explore molecular factors that may elucidate why HRD status does not consistently predict PARPi sensitivity. Therefore, we conducted a *post hoc* translational analysis of formalin-fixed paraffin-embedded tumor samples from the ENGOT-ov24/NSGO-AVANOVA part 1 and 2 trial (NCT02354131), focusing on alterations pertaining radiologic response and progression-free survival (PFS). DNA sequencing was performed using the TruSight Oncology 500 HT gene panel, with variants classified according to recent guidelines. HRD status had been assessed by Myriad MyChoice CDx. We identified, among 92 patients in the ENGOT-ov24/NSGO-AVANOVA part 1 and 2 trial, 151 pathogenic or likely pathogenic variants across 81 samples. PARPi-sensitizing variants were found in two out of 10 HRD-negative samples from patients with clinical benefit (PFS ≥12 months), whereas three out of 10 HRD-positive samples from patients having no benefit (PFS ≤6 months) harbored variants associated with PARPi resistance. Additionally, analysis of *BRCA1* variants revealed that truncating variants in exon 11 correlated with clinical benefit when niraparib was combined with bevacizumab. Conclusively, our findings highlight the complexity of PARPi response in AOC and underscore the importance of exploring somatic variants beyond HRD status. Further investigation into exon 11 variants of *BRCA1* and the potential of combination treatment is warranted.

**Significance:**

The irregular response to PARPi in HRD-positive and -negative tumors highlights the need for identifying additional biomarkers. This study explores the mutational landscape beyond HRD status in AOC, ultimately advancing precision oncology in future clinical practice.

## Introduction

The management of advanced epithelial ovarian cancer (AOC) has seen significant advancements through the use of molecular diagnostics and personalized treatment. Homologous recombination deficiency (HRD) status remains the most robust predictive biomarker for PARP inhibitor (PARPi) response. However, intriguingly, a subset of patients with HRD-negative (HRDneg) tumors still respond to treatment, whereas some patients with tumors classified as HRD-positive (HRDpos) do not benefit from PARPi.

The Myriad MyChoice CDx (Myriad Genetics, Inc.; MyChoice) and FoundationOne CDx (Foundation Medicine) tests stand out as FDA-approved tools for assessing HRD status and predicting patient responses to PARPi. Over the past years, several laboratory-developed HRD tests have emerged and undergone validation demonstrating concordance, particularly with the MyChoice test. Notably, the ENGOT European HRD initiative has played a crucial role in development and validation of the tests (1–5) using DNA samples from patients enrolled in the PAOLA-1 trial (6)—a phase III clinical trial investigating the addition of olaparib to bevacizumab as first-line maintenance treatment in ovarian cancer. Likewise, a laboratory-developed test for HRD status has been established at the Center for Genomic Medicine, Rigshospitalet. This laboratory-developed test aligns with the HRD status determined by the MyChoice test in patients enrolled in the ENGOT-ov24/NSGO-AVANOVA part 1 and 2 (AVANOVA1&2) trial conducted by NSGO-CTU (7).

Efforts in abrogating resistance remain a topic of great interest. Despite numerous proposed resistance mechanisms (8), a comprehensive understanding of these remains elusive. Preclinical studies have shown that antiangiogenic agents induce HRD in *BRCA1/2* wildtype (*BRCA1/2*wt) cells and in *BRCA1/2*-mutated (*BRCA1/2*m) cells who have become resistant to platinum agents (9). These findings support the combination of PARPi and antiangiogenics to be efficient, a regimen that has been further studied in two positive phase II studies (10, 11). In contrast, the phase III PAOLA-1 trial showed no progression-free survival (PFS) benefit by adding olaparib to bevacizumab in patients with HRDneg tumors regardless of several risk factors (12). Unfortunately, PAOLA-1 did not explore the effect of olaparib as monotherapy for comparison. Nevertheless, based on positive PFS results in the overall study population in the PRIMA study (13), niraparib has been approved as first-line maintenance therapy for all patients with newly diagnosed high-grade serous ovarian cancer regardless of HRD status.

Some clinical trials evaluating PARPi in AOC have published *post hoc* translational substudies, in which tissue samples from trial participants have been analyzed with next-generation sequencing (NGS) methods, mainly by the use of targeted gene panels (14–16). In the ARIEL2 study, *RAD51C/D* mutations and high-level *BRCA1* methylation predicted response to rucaparib similar to pathogenic variants in *BRCA1/2* (14). In the PAOLA-1 study, samples were filtered for all *BRCA1/2*m, and the authors found benefit of adding olaparib to bevacizumab irrespective of location of amino acid changes on both genes (15). Lheureux and colleagues (16) studied tumor tissue samples from Study 19 and Study 41 and identified *BRCA2* mutations enriched in long-term responders to olaparib.

In the present study, we sought to identify somatic alterations that could explain unexpected patient responses in terms of radiologic response and PFS in AVANOVA1&2.

## Materials and Methods

### Study design and participants

The ENGOT-ov24/NSGO-AVANOVA2 (NCT02354131) trial was an open-label, randomized, phase II study in which patients ages 18 years or older with measurable or evaluable [according to RECIST (version 1.1; ref. 17)] relapse of platinum-sensitive high-grade serous or endometroid ovarian cancer were randomized 1:1 to either once-daily oral niraparib 300 mg as monotherapy or in combination with intravenous bevacizumab 15 mg/kg once every 3 weeks until disease progression. A third treatment arm with bevacizumab given as monotherapy was closed at first amendment due to funding issues after the enrollment of six patients. Before initiation of the phase II study, a phase I study (ENGOT-ov24/NSGO-AVANOVA1) was conducted to assess the ideal dose of the combination regimen. Design and results of primary endpoints of both studies have previously been published (11, 18), and the studies are from now on referred to as AVANOVA1&2. The studies were conducted at 15 university hospitals in Denmark, Sweden, Finland, Norway, and the United States. Before enrollment to AVANOVA1&2, patients provided additional written informed consent to HRD testing used for stratification and subgroup analysis by MyChoice. Samples with genomic instability score (GIS) ≥42 and/or pathogenic variants in *BRCA1/2* were annotated as HRDpos. For the harmonization of clinical data from the phase I and II study, PFS is defined as time from first dose of niraparib until progression (and not from randomization to progression as stated in the protocol and reported in the publication from AVANOVA2). Patients with PFS ≥12 months were defined as having clinical benefit, also referred to as “responders,” and patients with PFS ≤6 months were defined as having no benefit, also referred to as “nonresponders.” Patients with PFS 6 to 12 months were considered in a gray zone as neither responders nor nonresponders. The present translational study consists of *post hoc* exploratory analysis of data from the AVANOVA1&2 and was conducted in accordance with the Declaration of Helsinki. An amendment to the AVANOVA1&2 trial protocol (H-1-2014-135) describing additional sequencing analysis has been approved by local institutional review boards or independent ethics committees of all investigational sites. Patients have provided a written informed consent to participate in translational research with a waiver of terminally ill or deceased patients. All patients included in AVANOVA1&2, except for the patients enrolled in the “bevacizumab as monotherapy”-arm, were eligible for *post hoc* translational analysis.

### DNA sequencing and variant filtering

Archival formalin-fixed paraffin-embedded (FFPE) tumor tissue, predominantly from each patient’s primary surgery, and preferably the same FFPE block that were used for HRD testing before entering the AVANOVA1&2 clinical trial, was collected from the participating university hospitals’ departments of pathology. At Center for Genomic Medicine, Rigshospitalet, DNA was extracted from the FFPE tissue using the QIAsymphony GeneRead DNA FFPE Treatment kit (Qiagen) and the Maxwell RSC instrument. The extracted DNA was quantified using the Qubit Fluorometer (Thermo Fisher Scientific). NGS analysis was performed on DNA libraries prepared from a minimum of 200 ng DNA. Libraries were hybridized using the TruSight Oncology 500 (TSO500) HT gene panel (Illumina) and sequenced on the NovaSeq 6000 to a minimum median coverage of 250×. Sequencing reads were processed, and variants were called using the TSO500 Illumina pipeline (Version: ruo-2.2.0.12). Covered genes by the TSO500 panel are provided in the Supplementary gene list S1. Variants present in ≥5% of the sequencing reads and in ≤1% of the background population (reported by gnomAD v2.1.1, RRID: SCR_014964) were assessed using the Qiagen Clinical Insight Interpret Translational software (Qiagen).

### Variant classification

Variants passing filtration settings were classified using the recommendations published by Horak and colleagues (19) for “classification of pathogenicity of somatic variants in cancer (oncogenicity).” A score of 6 or above corresponds to “likely oncogenic” and “oncogenic” variants, hereafter termed as *pathogenic* variants. Amplifications >5 fold-change were listed as *pathogenic*. In addition to the abovementioned classification scheme, variants in *BRCA1/2* were subsequently curated using the ENIGMA Classification Criteria from August 2023. Transcripts NM_007294.4 and NM_000059.4 were used for *BRCA1* and *BRCA2*, respectively. For all variants passing filtrations and classification criteria, both transcript version, sequencing depth, allele frequency, nucleotide, and amino acid substitution were curated upon manual verification using Integrative Genomics Viewer (RRID: SCR_011793).

### Data visualization and statistical analysis

**Figure 1:**
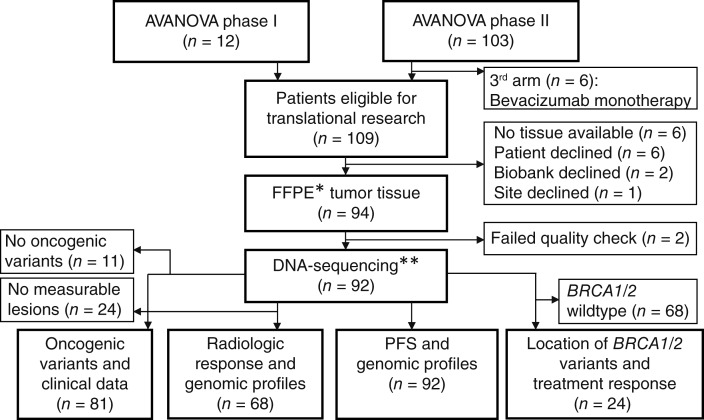
Flowchart showing patients included in final gene panel analysis. * Formalin-fixed paraffin-embedded. ** TSO500 gene panel by Illumina.

Illustrations and statistical analysis were generated using R version 4.3.0 (R Foundation for Statistical Computing, RRID: SCR_001905). Figure 1 was created in PowerPoint (Microsoft Corporation, RRID: SCR_023631) and lollipop plots of *BRCA1* and *BRCA2* were created in IBS 2.0 (20). Fisher’s exact test was applied to look for enriched alterations in patients with or without clinical benefit. Kaplan–Meier methodology was used to estimate PFS curves, and Cox’s proportional hazards model was used to compare subgroups with any *P* value <0.05 considered significant.

### Data availability

The data generated in this study are publicly available in both European Variation Archive and European Nucleotide Archive, via accession ID: PRJEB82346.

## Results

### Study cohort

Upon exclusion of six patients, who were included before the closure of the third treatment arm involving bevacizumab as monotherapy, a total of 109 patients remained eligible for subsequent translational research. Due to factors such as missing tissue, patient refusal, and non-participation from sites or regional biobanks, we successfully collected 94 FFPE tumor tissue samples of which 92 samples passed the quality check (Fig. 1). Patient characteristics were analyzed to assess the distribution of samples collected from each treatment arm – Table 1. Among the 92 samples, 41 originated from patients treated with niraparib as monotherapy, whereas 51 samples were from patients in the combination therapy arm. Despite this initial imbalance between the treatment arms, we found that the two groups were well-matched in terms of neoadjuvant chemotherapy, Federation Internationale des Gynaecologistes et Obstetristes (FIGO) stage, age, histology, performance status, and HRD status. Pathogenic variants in *BRCA1* were overrepresented in the monotherapy arm (*n* = 9/13) and *BRCA2* in the combination therapy arm (*n* = 8/11).

**Table 1:** Patient characteristics

Characteristic	Overall, *n* = 92	Combination, *n* = 51	Niraparib, *n* = 41
Median age at treatment start	66 (43–86)	67 (45–82)	66 (43–86)
Median age at primary surgery	63 (42–84)	64 (43–77)	62 (42–84)
Unknown	1	0	1
FIGO stage			
IIA	1	0	1
IIB	2	1	1
IIIA	3	2	1
IIIB	1	0	1
IIIC	54	31	23
IV	27	15	12
Unknown	4	2	2
Histologic subtype			
High-grade	85	47	38
Low-grade	5	3	2
Unknown	2	1	1
Performance status			
0	70	41	29
1	20	10	10
2	2	0	2
No. of prior lines of chemotherapy			
1	56	35	21
2	29	12	17
3	5	3	2
4	1	0	1
8	1	1	0
Neoadjuvant platinum treatment			
Yes	30	16	14
No	62	35	27
HRD status (MyChoice)			
Positive	49	27	22
Negative	34	20	14
Unknown	9	4	5
*BRCA1/2* mutations			
*BRCA1*m	13	4	9
*BRCA2*m	11	8	3
*BRCA1/2*wt	68	39	29

Abbreviation: FIGO, Federation Internationale des Gynaecologistes et Obstetristes.

### Samples suitable for gene panel profiling

We manually identified and confirmed pathogenic variants in 81 of 92 FFPE samples. Eleven samples did not reveal any pathogenic variants. Notably, two of the 11 samples were already diagnosed with a germline *BRCA1* and *BRCA2* pathogenic variant, respectively, as part of the initial AVANOVA1&2 trial conduction. Both germline variants were reidentified in the FFPE samples. To explore tumor purity in the 11 samples with unexpected lack of pathogenic variants, corresponding copy number array data, already generated as a part of another study from our group (7), was assessed. This assessment confirmed the suspicion of a high ratio of normal tissue in nine of the 11 samples including the two *BRCA1/2* germline samples, by revealing a balanced allele profile without copy number changes. Additionally, MyChoice HRD status was only successfully analyzed in five out of the nine samples. Among these, the two samples exhibiting *BRCA1/2* (germline) variants were categorized as HRDpos and three *BRCA1/2*wt samples resulted in GIS ranging from 8 to 18. The remaining four patients were stratified as HRDneg/unknown. MyChoice failed to analyze five additional samples within our study cohort, resulting in a total failure rate accounting for 10% (9/92 samples).

### BRCA1/2 mutation status

All pathogenic and likely pathogenic *BRCA1/2* variants identified through the initial MyChoice analysis were confirmed in the present gene panel results. Based on updated classification scheme, two variants in *BRCA1* and *BRCA2* were likely pathogenic; however, previously, they had been regarded as “variants of uncertain specificity.” The up-classification would not have had any consequence on stratification as they were both initially stratified as HRDpos based on the GIS alone. In addition, two *BRCA1* and two *BRCA2* variants of uncertain specificity were in fact likely benign variants based on updated criteria.

### Pathogenic variants and clinical data

**Figure 2:**
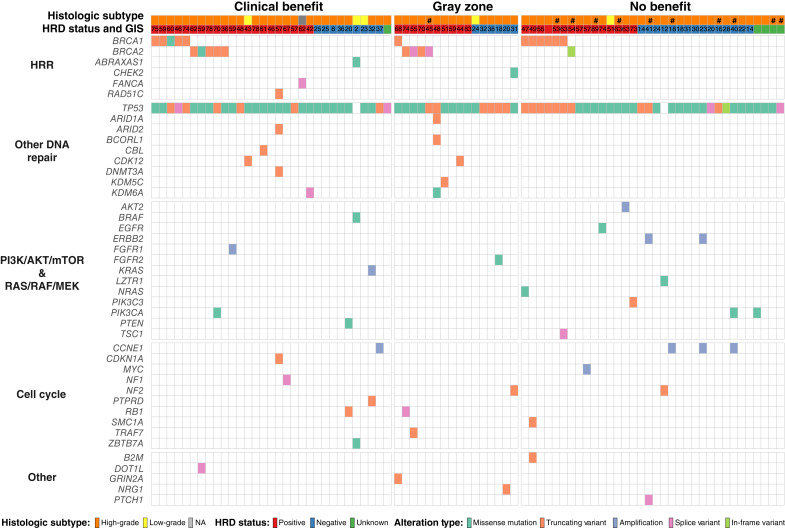
Oncoplot showing pathogenic gene alterations. The plot is split into groups by cellular pathways affected by the alteration on the vertical axis and clinical outcome (clinical benefit: PFS ≥12 months, no benefit: PFS ≤6 months) on the horizontal axis. Each column represents one patient. In total, 81 patient samples. See color guide reflecting specific alterations below the plot. # = Reason of “end of treatment” other than progressive disease. Integer “HRD-status and GIS” = the exact GIS reported by MyChoice.

In our study, we identified 151 pathogenic or likely pathogenic variants across the 81 tumor tissue samples – Fig. 2. Consistent with existing literature, deleterious mutations in *TP53* were present in nearly all high-grade serous AOC tumors (21). In total, 99% (*n* = 74/75) of the tumor tissue samples from high-grade serous AOC harbored a pathogenic variant in *TP53*. Notably, among the two *TP53*wt samples, one was associated with a low-grade serous AOC tumor harboring a *BRAF* V600E variant. Remarkably, pathogenic variants in *BRCA1/2* were present in patients both with and without clinical benefit, whereas variants in other known homologous recombination repair (HRR) genes were identified exclusively in tumor samples from patients with clinical benefit (*ABRAXAS1*, *FANCA*, and *RAD51C*). Additionally, pathogenic variants in other known DNA repair genes (excluding *TP53*) were significantly enriched in samples in which patients had obtained clinical benefit (*P* = 0.02). Interestingly, pathogenic variants in the PI3K/AKT/mTOR pathway and the RAS/RAF/MEK pathway were more prevalent among patients with no benefit (*n* = 10/34) than patients with clinical benefit (*n* = 5/31), although this difference was not statistically significant (*P* = 0.25). Pathogenic and likely pathogenic variants except for amplifications are listed in SupplementaryGeneTable S1.

### Radiologic response and overall HRD status

**Figure 3:**
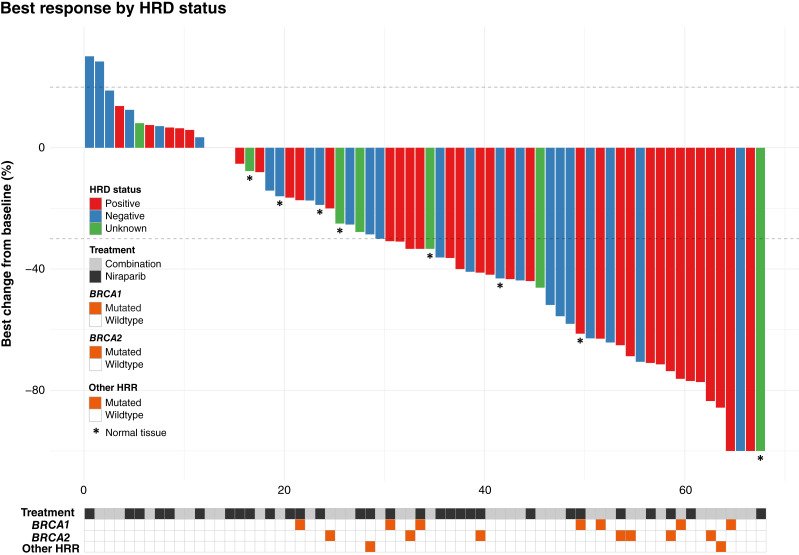
Best response in target lesions from baseline (RECIST 1.1-criteria). Dashed lines at +20% and −30% change from baseline equals stable disease. See guide in plot for color explanations.

AVANOVA1&2 was a clinical study in which patients were treated directly at relapse; therefore, all patients had measurable disease at inclusion. Measurable target lesions were evaluable in 70 patients, and in 68 of them “best radiologic response” could be calculated as minimum sum of target lesions across evaluation scans divided by sum of target lesions at baseline. Two of 70 patients had progressive disease at first scan with new lesions and are not reported here. Figure 3 visualizes best radiologic response annotated by HRD status. Notably, patients with HRDpos tumors were more likely to experience deep radiologic responses regardless of treatment arm. Additionally, no pathogenic variants in *BRCA1/2* or in other HRR genes were present in samples from patients who had radiologic tumor growth as best response. Supplementary Figures S1 and S2 illustrate calculated GIS for the patients with HRDneg tumor samples who had a radiologic shrinkage of tumor lesions in each treatment arm as well as for the patients with HRDpos samples with no tumor shrinkage. None of these unexpected responses could be explained by “borderline GIS,” meaning that the HRDneg tumors were not close to the cutoff at 42, except for one in the combination arm with complete response (GIS = 38) and one in the monotherapy arm with partial response (GIS = 37). Likewise, the HRDpos tumors without any tumor shrinkage all had high scores. Supplementary Figure S3 shows the two treatment arms’ best responses in direct comparison (NB: not the same number of patients in each plot).

### HRDpos tumors and radiologic response

For the 12 patients who were stratified as HRDpos and experienced stable disease as best radiologic response, three tumor samples had a competitive *gain-of-function* alteration in an oncogene (missense mutation in *EGFR*, amplification of *MYC* and *AKT2*), and two samples had a pathogenic variant in a tumor suppressor gene (a frameshift mutation in *KDM5C* and a predicted splice variant of *TSC1*, respectively). Reduced sensitivity to PARPi is described in the literature for *gain-of-function* variants in *EGFR* (22). A combination of MYC blockade with PARPi yielded synthetic lethality in *MYC*-driven triple-negative breast cancers (23), indicating a potential treatment opportunity for future clinical trials in AOC. Similarly, activation of the PI3K–Akt pathway is also associated with resistance to PARPi (24).

### HRDneg tumors and radiologic response

In contrast, only three out of 11 patients with HRDneg samples who experienced partial response, or complete response as best response had a concurrent pathogenic variant other than in *TP53*, and none of these variants (*CCNE1* amplification, frameshift mutation in *NRG1*, and missense mutation in *FGFR2*) could explain the paradoxical radiologic tumor shrinkage. All samples containing tumor tissue with unknown HRD status harbored a pathogenic *TP53* variant and no other pathogenic variants.

### PFS and overall HRD status

**Figure 4:**
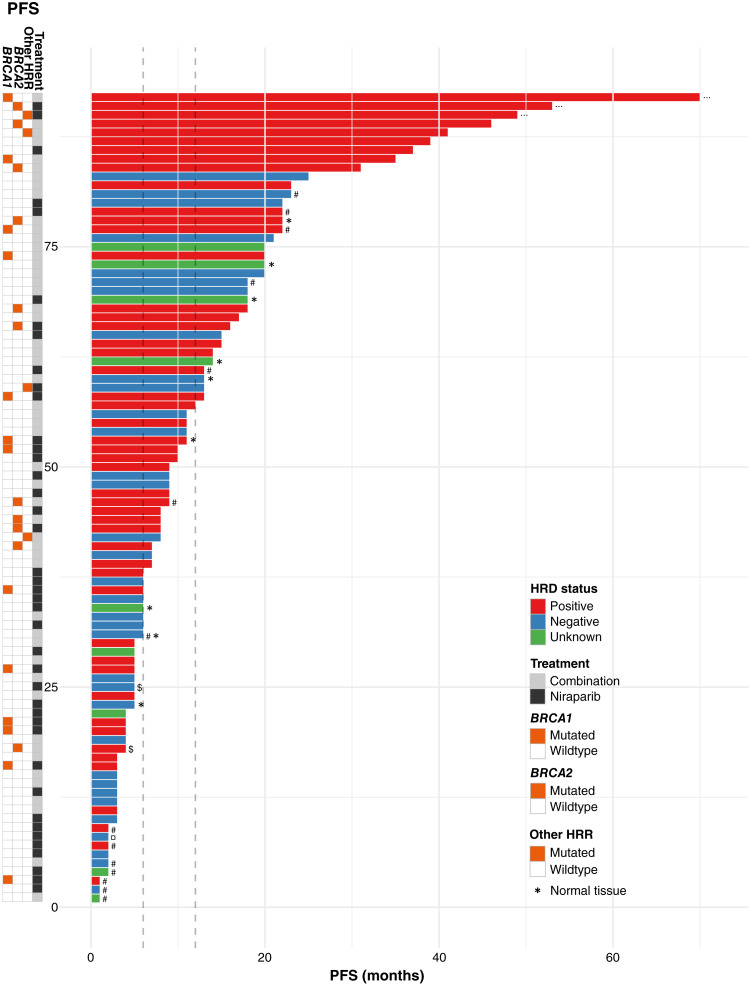
PFS in months colored by HRD status. Dashed lines at 6 and 12 months illustrate cutoff for “no benefit” and “clinical benefit”. *X*-axis indicates the number of patients. Reason for end of treatment other than progression: # = toxicity, $ = partial withdrawal of consent (study assessments continued), ¤ = investigator decision, and “…” = no progression at study closure. See guide in plot for color explanations.

PFS was the primary endpoint in AVANOVA1&2 and found that combination therapy was superior to monotherapy. Here, we report PFS for each patient annotated by HRD status (Fig. 4)*.* Among the 92 patients evaluable for PFS analysis, 17 patients stopped treatment for other reasons than progressive disease [toxicity (*n* = 14), partial withdrawal of consent (*n* = 2), investigator decision (*n* = 1)], and three patients were still on treatment at study closure. PFS separated on treatment arms with reported GIS for HRDneg stratified responders and HRDpos stratified nonresponders is illustrated in Supplementary Figs. S4 and S5*.* A direct comparison of the two treatment arms with regard to PFS is illustrated in Supplementary Fig. S6. For the patient cohort included in this study, we conducted PFS analysis using Kaplan–Meier methodology and Cox proportional hazards calculation (Supplementary Figs. S7–S10). Notably, patients with unknown HRD status were excluded from the analysis in contrast to the primary publication, in which the patients were analyzed as HRDneg/unknown (11). Within this subcohort, there was no statistically significant difference in PFS between patients who received combination therapy and monotherapy (*P* = 0.18; Supplementary Fig. S10). However, a significant PFS benefit was identified in patients with HRDpos samples when not considering the two treatment arms (HR = 0.56; 95% confidence interval, 0.34–0.95; *P* = 0.03; Supplementary Fig. S7).

### HRDpos tumors and PFS

Among 10 patients with HRDpos samples, who progressed before 6 months of treatment, five had a pathogenic *BRCA1* variant, all in combination with a concomitant *TP53* mutation. Three of the five nonresponding patients with a pathogenic *BRCA1* variant had a concurrent pathogenic variant in at least one other gene found by targeted sequencing (*NRAS*, *SMC1A*, *B2M*, and *TSC1*). These genes in combination with a *BRCA1* mutation have so far not been reported to confer resistance to PARPi. For the remaining five patients with HRDpos tumors, who progressed within 6 months, three had a concurrent pathogenic variant besides a *TP53*m (*EGFR*, *MYC*, and *AKT2*). These refer to the same three patients previously described in terms of radiologic response.

### HRDneg tumors and PFS

Of 10 patients stratified as HRDneg, who experienced clinical benefit (regardless of reason for stopping treatment), nine had pathogenic variants, among them five samples revealed pathogenic variants only in *TP53*. One sample was found to be from a low-grade tumor with pathogenic variants in *ZBTB7A*, *ABRAXAS1*, and *BRAF*, and no pathogenic variants in *TP53*. Another sample had pathogenic variants in *RB1* and *PTEN*. *ZBTB7A*, *ABRAXAS1*, and *RB1* are reported to be associated with response to PARPi and/or platinum treatment (25–28); conversely, *PTEN* has conflicting interpretations with regard to PARPi sensitivity (29–31). The two remaining HRDneg samples revealed amplification of *KRAS* together with a truncating variant in *PTPRD*, and amplification of *CCNE1*, respectively, with both amplifications associated with resistance to PARPi (32, 33). Despite the identification of both sensitizing and resistance inducing alterations beyond HRD status, it is still remarkable, that among the nine patients with longest PFS, all had HRDpos tumors, and seven had deleterious variants in *BRCA1/2* or another HRR gene. The three patients still on treatment at study closure and with the longest PFS had truncating variants in *BRCA1*, *BRCA2*, or *RAD51C*, respectively.

### Location of BRCA1/2 variants and treatment response

**Figure 5:**
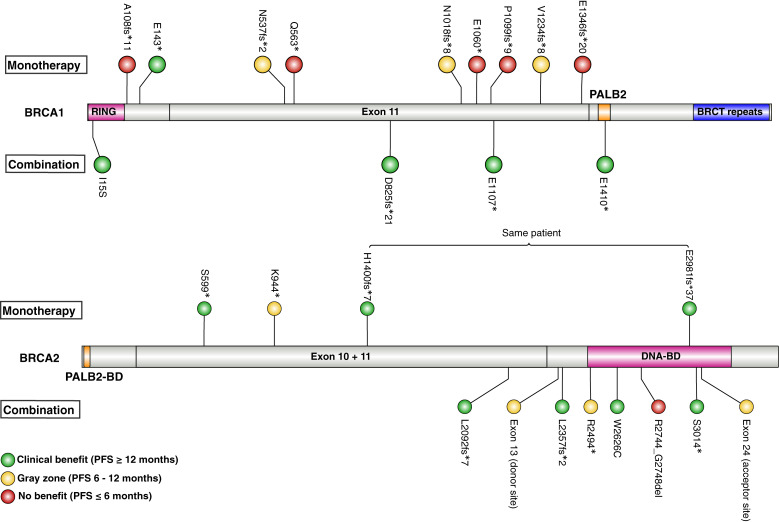
*BRCA1* (top) and *BRCA2* (bottom) with variants found in AVANOVA1&2 colored by treatment response (see legend). *BRCA1*: Transcript NM_007294.4. In total, 1,863 aa. RING domain (aa 2–101), *PALB2*-binding domain (also called coiled-coil domain; aa 1,391–1,424), BRCT repeats (aa 1,650–1857), and exon 11 (aa 224–1,366). *BRCA2*: Transcript NM_000059.4. In total, 3,418 aa. *PALB2*-binding domain (aa 10–40), DNA-binding domain (aa 2,481–3,186), and exons 10 and 11 (aa 266–2,281).

Pathogenic missense variants were observed only once in *BRCA1* (I15S) and *BRCA2* (W2626C) and were located in important functional domains, RING and DNA-binding, respectively. For both missense variants, clinical benefit was observed upon combination therapy. Two *BRCA2* splice variants in exon 13 (donor site) and exon 24 (acceptor site) were predicted by SpliceAI (34) to be deleterious but were not associated with a clinical benefit. However, truncating variants (either as nonsense or frameshift mutations) were the most common and were randomly distributed with no association between location and response. Interestingly, only two of nine truncating variants in exon 11 of *BRCA1* were associated with a clinical benefit – see Fig. 5 for illustration. This aligns with evidence suggesting alternative splicing of *BRCA1* in the presence of nonsense mutations in exon 11 resulting in the expression of a BRCA1-Δ11q isoform. BRCA1-Δ11q is associated with resistance to both PARPi and cisplatin (35). Among the seven truncating variants in *BRCA1* exon 11 without clinical benefit, five were classified as HRDpos by MyChoice, whereas HRD estimation in the remaining two samples failed analysis. The two patients who had an exon 11 truncating variant and experienced clinical benefit had combination therapy, and the patients without clinical benefit received niraparib as monotherapy. Despite the limited cases, this indicates a potential benefit for combination therapy for patients with pathogenic variants in exon 11 of *BRCA1* (*P* = 0.03). For the *BRCA2*m responders, two received monotherapy and four received combination therapy, and no significant difference was identified with regard to the variant location.

## Discussion

In this study, we pursued molecular findings that may explain why HRD status is not always predictive of PARPi sensitivity. Our cohort consisted of 92 patients treated for a platinum-sensitive relapse of AOC with either niraparib as monotherapy or in combination with bevacizumab. The study is limited by the number of patients included and the FFPE tissue available. Additionally, the patients received two different treatment regimens, which limits the numbers for comparison. However, among the 10 patients with HRDpos samples, who progressed within 6 months after initiation of treatment, seven had other *pathogenic* variants than *TP53*m, and three of the patients had variants (*gain-of function* alteration in *EGFR*, *MYC*, and *AKT2*) associated with PARPi resistance (22–24). Likewise, among the 10 patients with HRDneg tumors, who progressed after 12 months of treatment, two was found to have PARPi sensitizing alterations in *ABRAXAS1* and *ZBTB7A*, and in *RB1*, respectively (25–28). In contrast, two patients still experienced positive clinical outcomes, although they had HRDneg tumor samples with amplifications of *KRAS* and *CCNE1*, respectively, typically associated with resistance to PARPi (32, 33). If these alterations were assessed upfront, it would probably not have changed the course of treatment for the patients with HRDpos tumors. Thus, missing the chance of treating a potential responder due to an HRDneg tumor sample with PARPi could be diminished if a wider panel of genes were explored, revealing potential PARPi-sensitizing variants. In that case, two out of 34 patients with HRDneg tumors in this study could have been detected as potential responders. Another interesting finding is the few responders with truncating variants in exon 11 of *BRCA1*, and that these responders received combination therapy and not monotherapy. A cautious interpretation of this could be that patients with *BRCA1* exon 11 variants potentially need combination therapy with, i.e., bevacizumab to sensitize tumor cells to PARPi. To further explore this hypothesis, we plan to analyze PARPi responses in patients with *BRCA1/2* variants in a wider cohort as next step. Costs versus effectiveness is an ongoing issue when adding high-cost tests (e.g., NGS analysis) to routine diagnostics. Considering the revolutionary era of genomic profiling along with rapidly declining sequencing costs, it is highly likely that the number of somatic cancers undergoing high-throughput sequencing analysis is increasing. Therefore, including comprehensive cancer variant mapping as exploratory translational studies to existing clinical trials is highly relevant when generating new hypothesis.

The high failure rates when using FFPE tumor tissue as input for MyChoice testing leave a gap for those patients having an inconclusive result. In this study, the MyChoice failure rate was 10% compared with ∼20% found in real-world routine MyChoice testing (36). The high failure rates are mainly due to poor DNA quality and/or low tumor cellularity. Ongoing studies aim to find better input tissue i.e., the use of ctDNA from peritoneal fluid. As academic HRD tests are being validated and published as open-source scripts, some of the tests have other sequencing and algorithm approaches than Myriad MyChoice and FoundationOne CDx and could potentially be used on other input tissue specimens than FFPE. Two studies are published showing encouraging results on ctDNA from peritoneal fluid (37, 38).

Worldwide, commercially available HRD tests are being used for determining the best treatment for patients with AOC, although PARPis are approved as first-line maintenance treatment by the European Medicines Agency and FDA regardless of HRD status. Costs and potential side effects are to be considered if treating a patient with a lower chance of benefit, such as those with HRDneg tumors. Another treatment option as first-line maintenance therapy for these patients could be bevacizumab as monotherapy (6). AVANOVA part 2 explored the use of niraparib as monotherapy or in combination with bevacizumab as treatment for relapse of ovarian cancer with a benefit in the combination arm (11). With careful interpretation, it seemed that adding bevacizumab to niraparib added a little extra to the tumor shrinkage effect in the cohort studied here, when treated directly at relapse. Even though the PAOLA-1 trial did not find a benefit of the combination of olaparib and bevacizumab in patients stratified as HRDneg in a first-line maintenance setting, the NIRVANA-1 trial currently recruits patients in another phase II study to receive niraparib or niraparib in combination with bevacizumab after adjuvant chemotherapy to further explore the benefit of adding an antiangiogenic agent to PARPi (39).

To the best of our knowledge, this is among the first few translational substudies to clinical trials in ovarian cancer pursuing individual explanations for PARPi sensitivity in patients with HRDneg tumors and PARPi resistance in patients with HRDpos tumors. We suggest further investigation into exon 11 variants of *BRCA1* to explore if some of these tumors should be treated differently, i.e., with combination therapy, to obtain response to PARPi.

More functional studies in cell cultures and retrospective genomic analysis of patients who have received PARPi treatment in clinical trials are warranted. Finally, exploiting and combining modalities beyond genomic sequencing, such as transcriptomics, proteomics, epigenetic, and spatial analysis, potentially unravels deeper insight into PARPi sensitivity. Taken together, identifying additional components involved in the response and resistance to PARPis is crucial to further enhance the personalized treatment of patients with AOC.

## Supplementary Material

Supplementary Material Supplementary Material

SupplementaryGeneTable S1 All identified pathogenic variants in the samples analyzed.
