# Supplementary material for: Beyond HRD Status: Unraveling Genetic Variants Impacting PARP Inhibitor Sensitivity in Advanced Ovarian Cancer
Source: Cancer Res Commun. 2024 Dec 26;4(12):3190–200. doi: 10.1158/2767-9764.CRC-24-0294 (PMC11670052; doi:10.1158/2767-9764.CRC-24-0294)
Supplement: Supplementary Material [file crc-24-0294_supplementary_material_suppsm.docx]

### Supplementary material


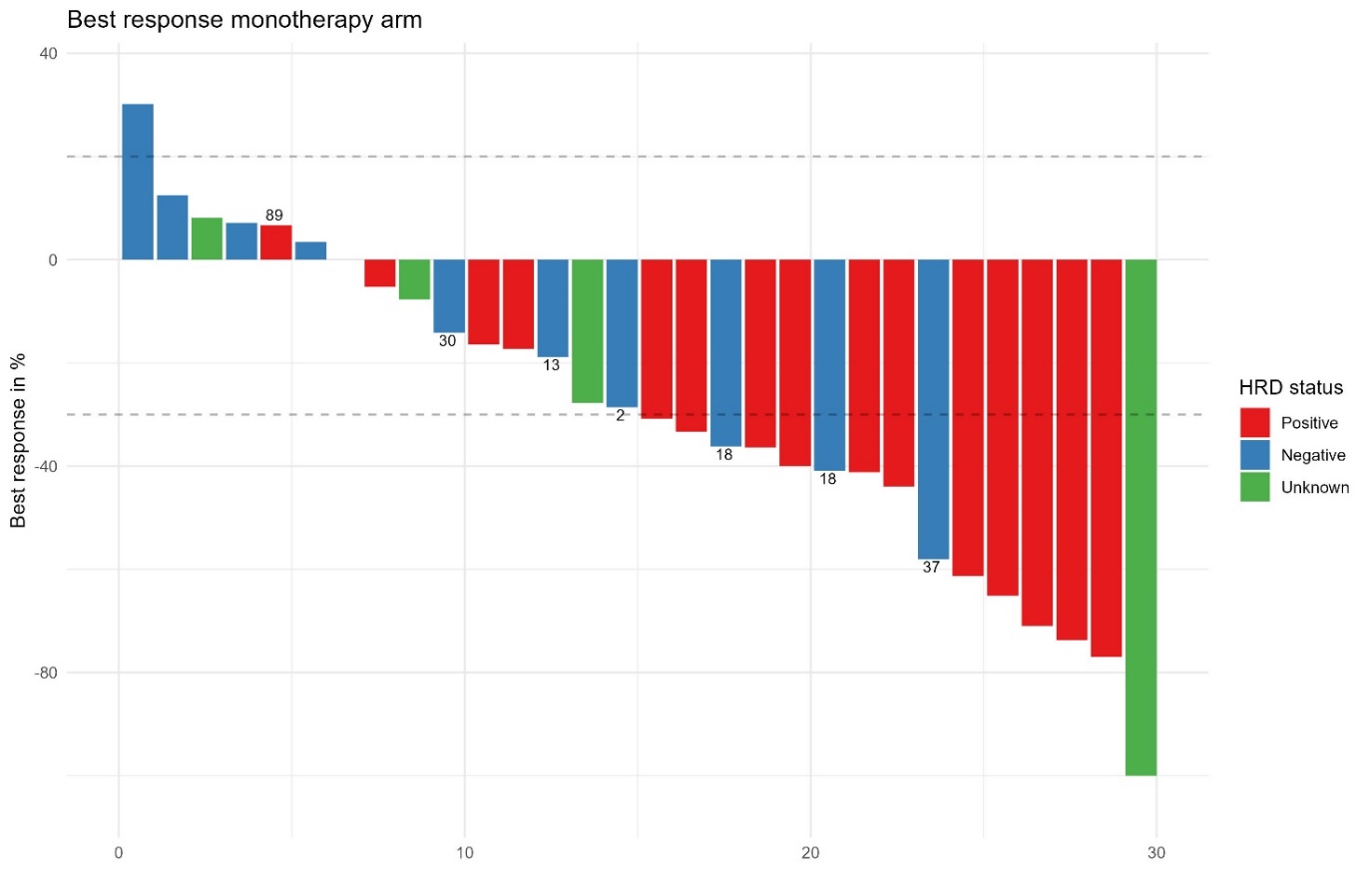


Supplementary figure S1: Radiological response in the monotherapy arm with reported GIS in HRDneg tumors with tumor shrinkage, and in the HRDpos tumor with tumor growth as best response.


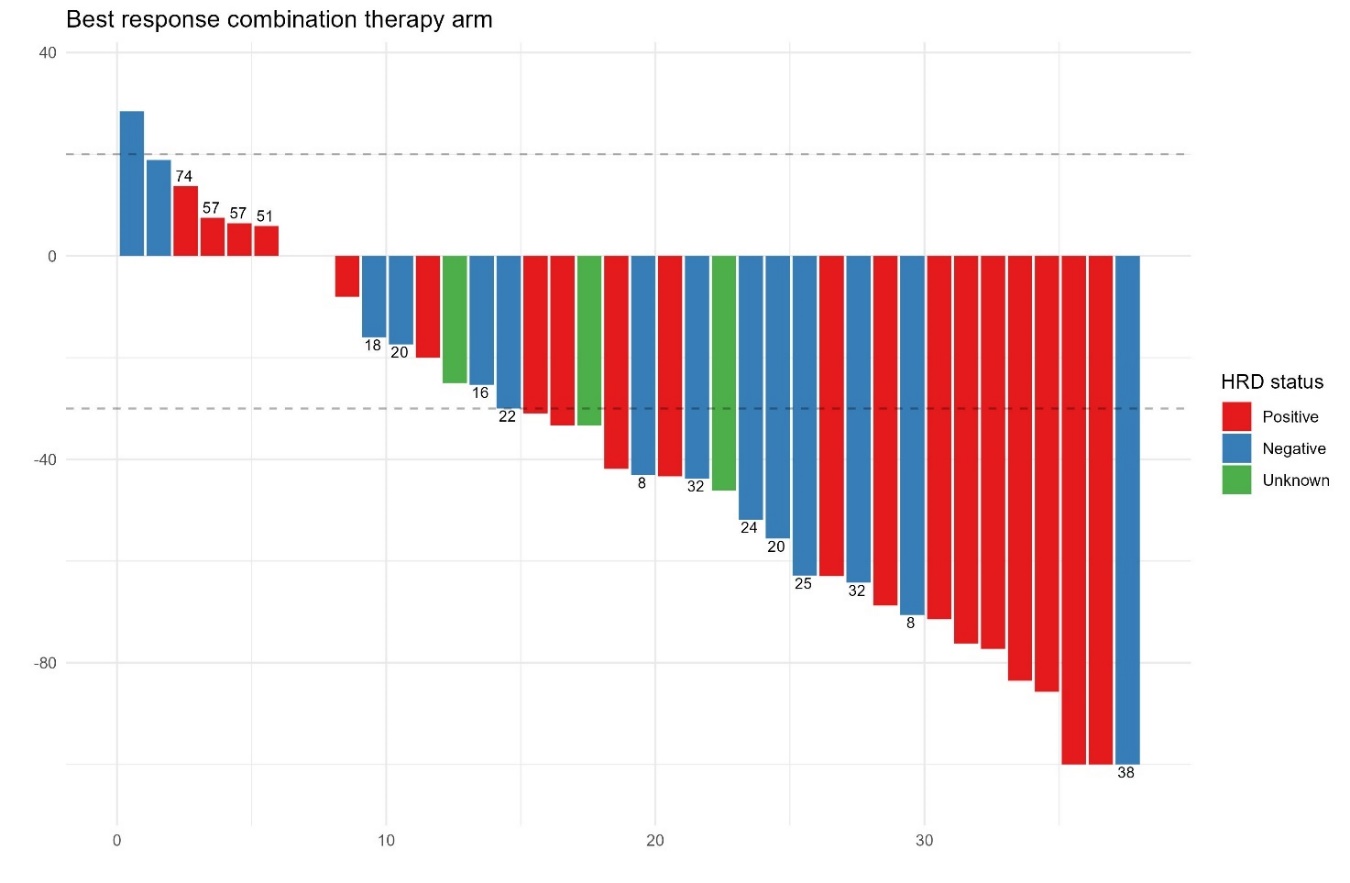


Supplementary figure S2: Radiological response in the combination arm with reported GIS in HRDneg tumors with tumor shrinkage, and in HRDpos tumors with tumor growth as best response.


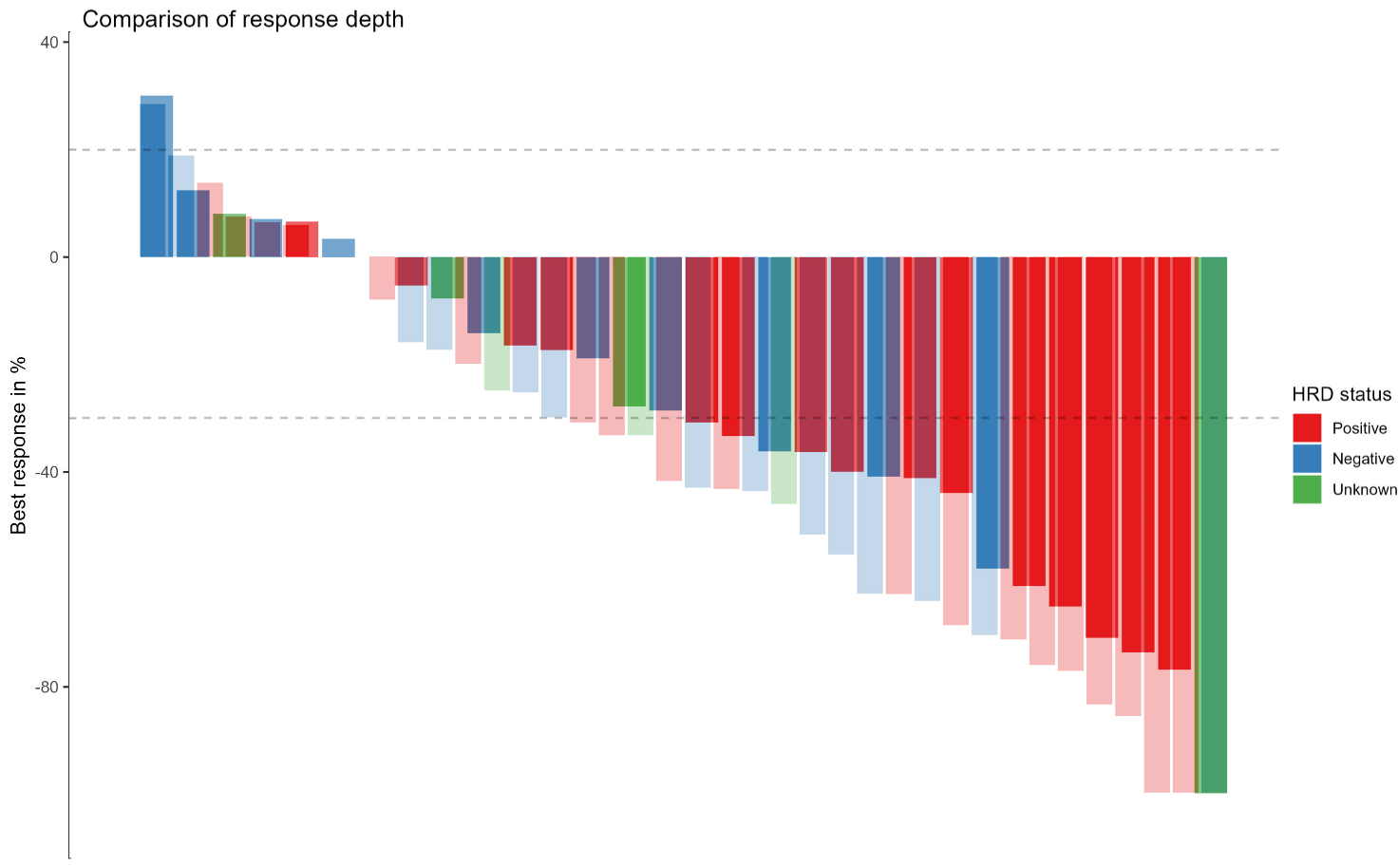


Supplementary figure S3: Radiological response in direct comparison of the two treatment arms. NB: there are not the same number of patients in each treatment arm.


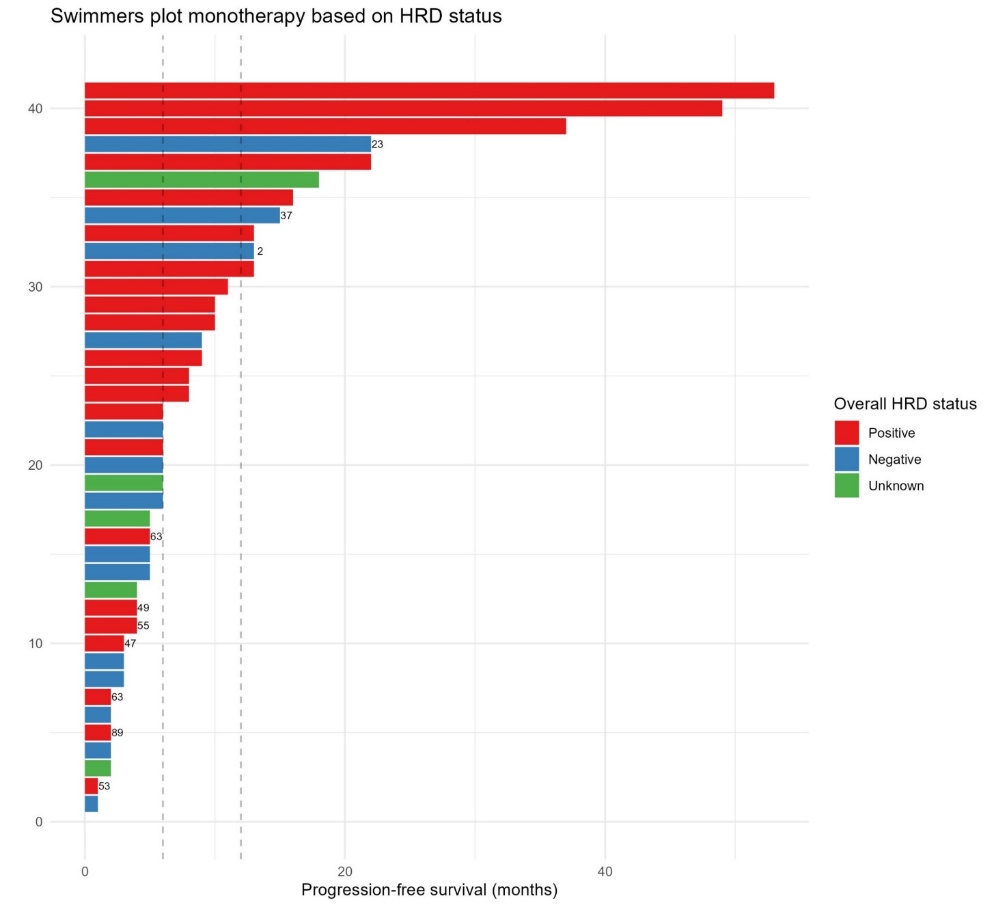


Supplementary figure S4: PFS in the monotherapy arm with reported GIS in HRDpos tumors from patients with no benefit, and in HRDneg tumors from patients with clinical benefit.


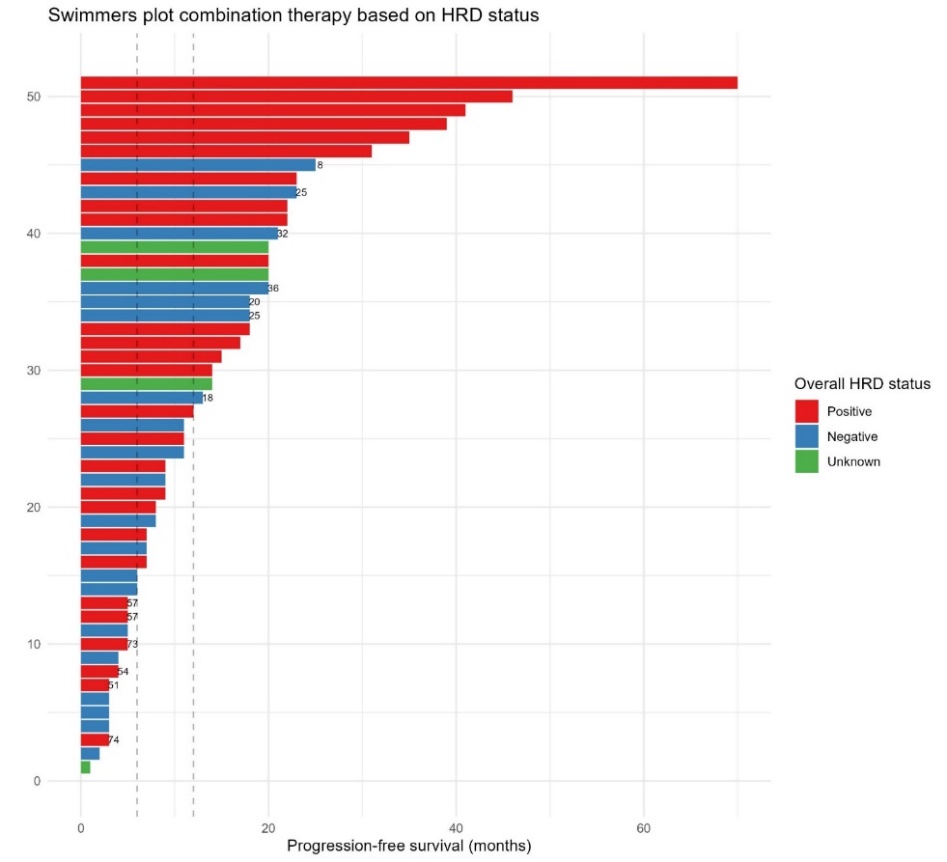


Supplementary figure S5: PFS in the combination therapy arm with reported GIS in HRDpos tumors from patients with no benefit, and in HRDneg tumors from patients with clinical benefit.


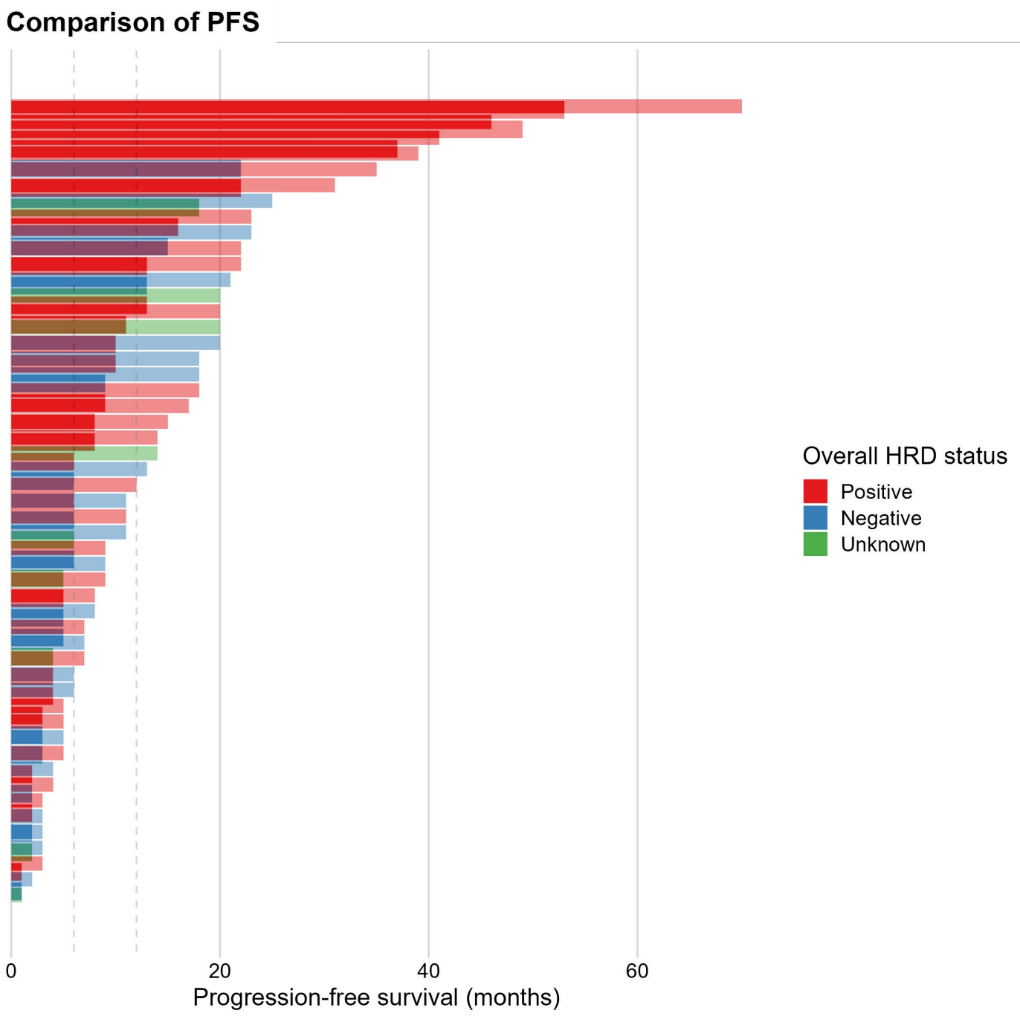


Supplementary figure S6: PFS in direct comparison of the two treatment arms. NB: there are not the same number of patients in each treatment arm.


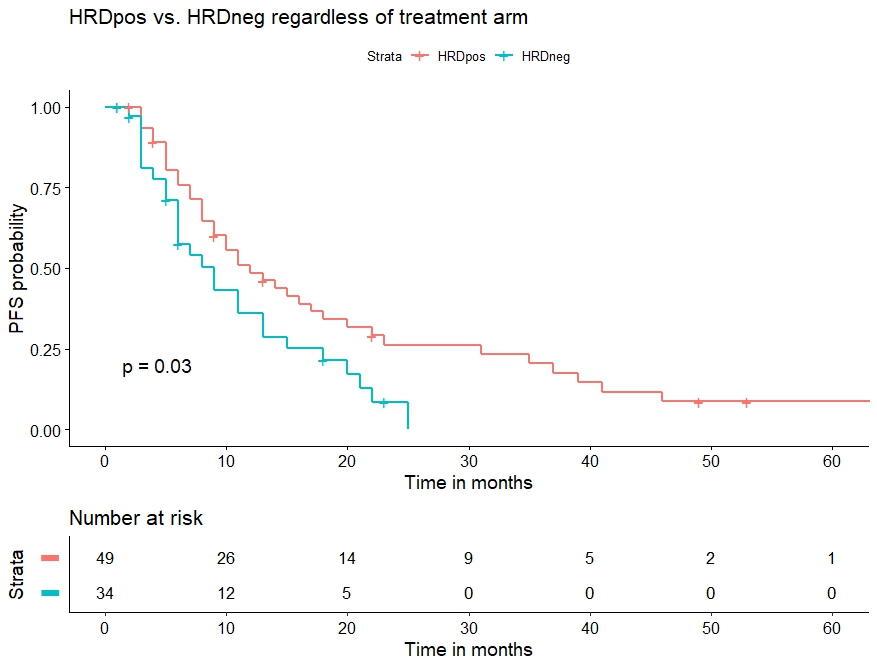


Supplementary figure S7: Progression-free survival of HRDpos vs. HRDneg regardless of treatment arm.
HR = 0.56 (95%CI 0.34 - 0.95, p = 0.03)


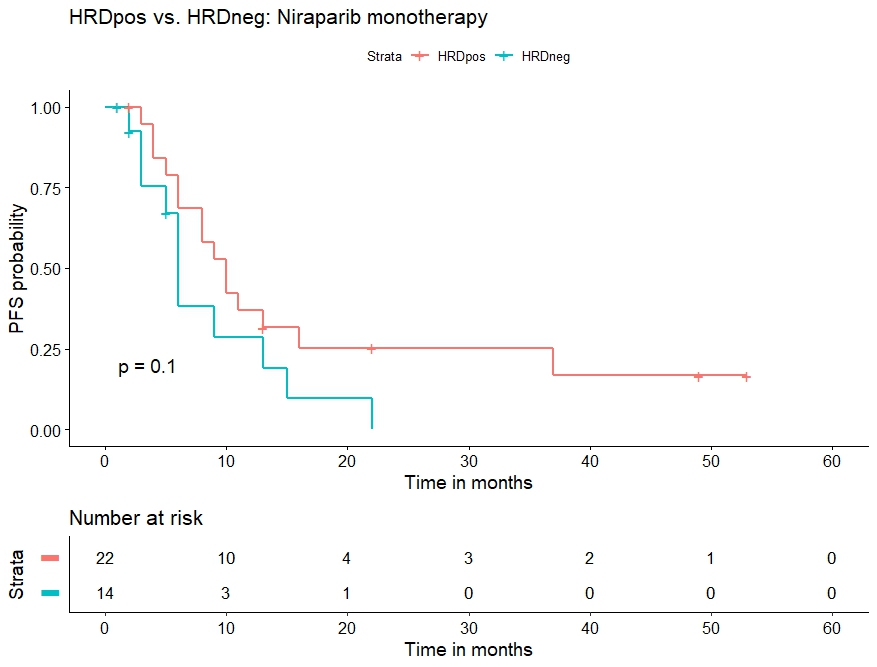


Supplementary figureS8: Progression-free survival of HRDpos vs. HRDneg in the niraparib monotherapy arm.
HR = 0.52 (95%CI 0.23 - 1.1, p = 0.1)


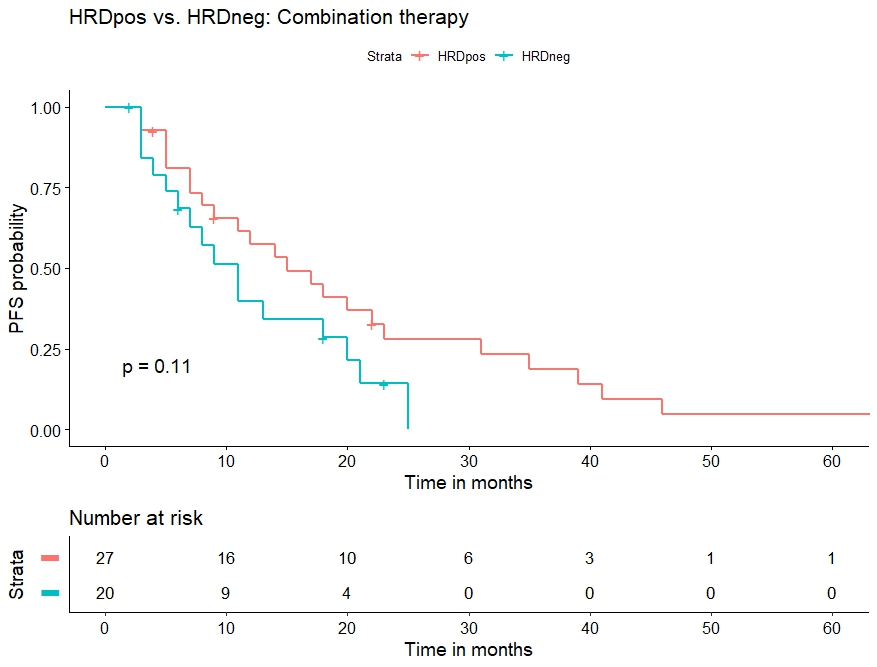


Supplementary figure S9: Progression-free survival of HRDpos vs. HRDneg in the niraparib+bevacizumab combination therapy arm.
HR = 0.58 (95%CI 0.29 - 1.1, p = 0.11)


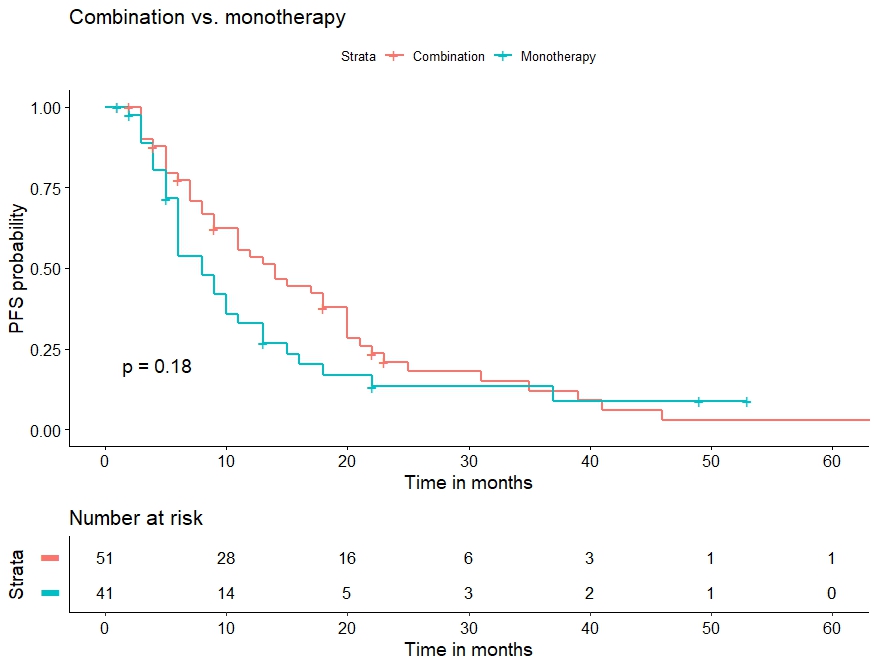


Supplementary figure S10: Progression-free survival of the two treatment arms in comparison.
HR = 0.072 (95%CI 0.45 - 1.2, p = 0.18)

### Supplementary gene list 1: TSO500 panel

ABL1 BCR CHEK1 EPHA7 FGF23 GSK3B IDH2 MAP3K1 NF2 PIK3CA RAD51D SMAD4 TGFBR2 ABL2 BIRC3 CHEK2 EPHB1 FGF3 H3F3A IFNGR1 MAP3K13 NFE2L2 PIK3CB RAD52 SMARCA4 TMEM127 ACVR1 BLM CIC ERBB2 FGF4 H3F3B INHBA MAP3K14 NFKBIA PIK3CD RAD54L SMARCB1 TMPRSS2 ACVR1B BMPR1A CREBBP ERBB3 FGF5 H3F3C INPP4A MAP3K4 NKX2-1 PIK3CG RAF1 SMARCD1 TNFAIP3 AKT1 BRAF CRKL ERBB4 FGF6 HGF INPP4B MAPK1 NKX3-1 PIK3R1 RANBP2 SMC1A TNFRSF14 AKT2 BRCA1a CRLF2 ERCC1 FGF7 HIST1H1C INSR MAPK3 NOTCH1 PIK3R2 RARA SMC3 TOP1 AKT3 BRCA2a CSF1R ERCC2 FGFR1 HIST1H2BD IRF2 MAX NOTCH2 PIK3R3 RASA1 SMO TOP2A ALK BRD4 CSF3R ERCC3 FGFR2 HIST1H3A IRF4 MCL1 NOTCH3 PIM1 RB1 SNCAIP TP53 ALOX12B BRIP1 CSNK1A1 ERCC4 FGFR3 HIST1H3B IRS1 MDC1 NOTCH4 PLCG2 RBM10 SOCS1 TP63 ANKRD11 BTG1 CTCF ERCC5 FGFR4 HIST1H3C IRS2 MDM2 NPM1 PLK2 RECQL4 SOX10 TRAF2 ANKRD26 BTK CTLA4 ERG FH HIST1H3D JAK1 MDM4 NRAS PMAIP1 REL SOX17 TRAF7 APC C11orf30 CTNNA1 ERRFI1 FLCN HIST1H3E JAK2 MED12 NRG1 PMS1 RET SOX2 TSC1 AR CALR CTNNB1 ESR1 FLI1 HIST1H3F JAK3 MEF2B NSD1 PMS2 RFWD2 SOX9 TSC2 ARAF CARD11 CUL3 ETS1 FLT1 HIST1H3G JUN MEN1 NTRK1 PNRC1 RHEB SPEN TSHR ARFRP1 CASP8 CUX1 ETV1 FLT3 HIST1H3H KAT6A MET NTRK2 POLD1 RHOA SPOP U2AF1 ARID1A CBFB CXCR4 ETV4 FLT4 HIST1H3I KDM5A MGA NTRK3 POLE RICTOR SPTA1 VEGFA ARID1B CBL CYLD ETV5 FOXA1 HIST1H3J KDM5C MITF NUP93 PPARG RIT1 SRC VHL ARID2 CCND1 DAXX ETV6 FOXL2 HIST2H3A KDM6A MLH1 NUTM1 PPM1D RNF43 SRSF2 VTCN1 ARID5B CCND2 DCUN1D1 EWSR1 FOXO1 HIST2H3C KDR MLL PAK1 PPP2R1A ROS1 STAG1 WISP3 ASXL1 CCND3 DDR2 EZH2 FOXP1 HIST2H3D KEAP1 MLLT3 PAK3 PPP2R2A RPS6KA4 STAG2 WT1 ASXL2 CCNE1 DDX41 FAM123B FRS2 HIST3H3 KEL MPL PAK7 PPP6C RPS6KB1 STAT3 XIAP ATM CD274 DHX15 FAM175A FUBP1 HLA-A KIF5B MRE11A PALB2 PRDM1 RPS6KB2 STAT4 XPO1 ATR CD276 DICER1 FAM46C FYN HLA-B KIT MSH2 PARK2 PREX2 RPTOR STAT5A XRCC2 ATRX CD74 DIS3 FANCA GABRA6 HLA-C KLF4 MSH3 PARP1 PRKAR1A RUNX1 STAT5B YAP1 AURKA CD79A DNAJB1 FANCC GATA1 HNF1A KLHL6 MSH6 PAX3 PRKCI RUNX1T1 STK11 YES1 AURKB CD79B DNMT1 FANCD2 GATA2 HNRNPK KMT2B MST1 PAX5 PRKDC RYBP STK40 ZBTB2 AXIN1 CDC73 DNMT3A FANCE GATA3 HOXB13 KMT2C MST1R PAX7 PRSS8 SDHA SUFU ZBTB7A AXIN2 CDH1 DNMT3B FANCF GATA4 IGF1 KMT2D MTOR PAX8 PTCH1 SDHAF2 SUZ12 ZFHX3 AXL CDK12 DOT1L FANCG GATA6 IGF1R KRAS MUTYH PBRM1 PTEN SDHB SYK ZNF217 B2M CDK4 E2F3 FANCI GEN1 IGF2 LAMP1 MYB PDCD1 PTPN11 SDHC TAF1 ZNF703 BAP1 CDK6 EED FANCL GID4 IKBKE LATS1 MYC PDCD1LG2 PTPRD SDHD TBX3 ZRSR2 BARD1 CDK8 EGFL7 FAS GLI1 IKZF1 LATS2 MYCL1 PDGFRA PTPRS SETBP1 TCEB1 BBC3 CDKN1A EGFR FAT1 GNA11 IL10 LMO1 MYCN PDGFRB PTPRT SETD2 TCF3 BCL10 CDKN1B EIF1AX FBXW7 GNA13 IL7R LRP1B MYD88 PDK1 QKI SF3B1 TCF7L2 BCL2 CDKN2A EIF4A2 FGF1 GNAQ INHA LYN MYOD1 PDPK1 RAB35 SH2B3 TERC BCL2L1 CDKN2B EIF4E FGF8 GNAS HRAS LZTR1 NAB2 PGR RAC1 SH2D1A TERTb BCL2L11 CDKN2C EML4 FGF9 GPR124 HSD3B1 MAGI2 NBN PHF6 RAD21 SHQ1 TET1 BCL2L2 CEBPA EP300 FGF10 GPS2 HSP90AA1 MALT1 NCOA3 PHOX2B RAD50 SLIT2 TET2 BCL6 CENPA EPCAM FGF14 GREM1 ICOSLG MAP2K1 NCOR1 PIK3C2B RAD51 SLX4 TFE3 BCOR CHD2 EPHA3 FGF19 GRIN2A ID3 MAP2K2 NEGR1 PIK3C2G RAD51B SMAD2 TFRC BCORL1 CHD4 EPHA5 FGF2 GRM3 IDH1 MAP2K4 NF1 PIK3C3 RAD51C SMAD3 TGFBR

Supplementary gene list: Genes covered by the targeted gene panel: TSO500
